# Synthesis of typical sulfonamide antibiotics with [^14^C]- and [^13^C]-labeling on the phenyl ring for use in environmental studies

**DOI:** 10.1186/s12302-022-00598-z

**Published:** 2022-03-08

**Authors:** Xuan Wu, Yao Yao, Lianhong Wang, Dashun Zhou, Feifei Sun, Jianqiu Chen, Philippe Francois-Xavier Corvini, Rong Ji

**Affiliations:** 1 State Key Laboratory of Pollution Control and Resource Reuse, School of the Environment, Nanjing University, Nanjing, 210023 China; 2 School of Engineering, China Pharmaceutical University, Nanjing, 211198 China; 3 Institute for Ecopreneurship, School of Life Sciences, University of Applied Sciences and Arts Northwestern Switzerland, Gründenstrasse 40, 4132 Muttenz, Switzerland

**Keywords:** Sulfonamides, Carbon-14, Carbon-13, Synthesis, Isotope tracer

## Abstract

**Background:**

Due to their widespread use, sulfonamide antibiotics (SAs) have become ubiquitous environmental contaminants and thus a cause of public concern. However, a complete understanding of the behavior of these pollutants in complex environmental systems has been hampered by the unavailability and high cost of isotopically labeled SAs.

**Results:**

Using commercially available uniformly [^14^C]- and [^13^C]-labeled aniline as starting materials, we synthesized [phenyl-ring-^14^C]- and [phenyl-ring-^13^C]-labeled sulfamethoxazole (SMX), sulfamonomethoxine (SMM), and sulfadiazine (SDZ) in four-step (via the condensation of labeled *N*-acetylsulfanilyl chloride and aminoheterocycles) or five-step (via the condensation of labeled *N*-acetylsulfonamide and chloroheterocycles) reactions, with good yields (5.0–22.5% and 28.1–54.1% for [^14^C]- and [^13^C]-labeled SAs, respectively) and high purities (> 98.0%).

**Conclusion:**

The synthesis of [^14^C]-labeled SAs in milligram amounts enables the preparation of labeled SAs with high specific radioactivity. The efficient and feasible methods described herein can be applied to the production of a variety of [^14^C]- or [^13^C]-labeled SAs for studies on their environmental behavior, including the fate, transformation, and bioaccumulation of these antibiotics in soils and aqueous systems.

**Supplementary Information:**

The online version contains supplementary material available at 10.1186/s12302-022-00598-z.

## Background

Sulfonamide antibiotics (SAs) are widely used in the treatment of human disease and in modern animal husbandry. However, due to their poor biodegradation and insufficient removal by wastewater treatment plants [1, 2], high concentrations of sulfadiazine (SDZ), sulfamethoxazole (SMX), and sulfamonomethoxine (SMM) are commonly detected in soils, sediments, rivers and other environmental media [3–5]. SAs that enter the environment exert adverse effects on organisms [6–9], thus raising public concern. A comprehensive understanding of the environmental fate of SAs, including their adsorption, biodegradation, transformation, formation of non-extractable residues (NERs), and transport, is essential to assessing their environment risks.

Studies of the environmental behavior of pollutants often rely on the use of [^14^C]-radioactive and [^13^C]-stable isotopes. The advantages of [^14^C]-tracers include their low detection limit and convenient handling with complex environmental samples. Consequently, they are frequently used to investigate the environmental fate of organic pollutants, especially mineralization and NERs formation. For example, [^14^C]-tracers have been employed to examine the environmental impacts of pesticides, brominated flame retardants, alkylphenols, and polycyclic aromatic hydrocarbons [10–14]. Stable isotopes (e.g., ^13^C, ^15^ N) have been used in mass spectrometry and nuclear magnetic resonance (NMR) spectroscopy studies to quantify and identify metabolites of pollutants in complex matrices [15–20]. [^13^C]-tracers provide powerful tools for investigations of microbial biomass and community composition and have thus been used in phospholipid fatty acid analyses and as DNA stable-isotope probes [21, 22]. This wide range of applications has increased the demand for [^14^C]- and [^13^C]-labeled SAs, but these isotopes are either commercially unavailable or too expensive. An efficient, simple method allowing the ‘‘in house’’ synthesis of [^14^C]- and [^13^C]-SAs, especially on micro-scales with good yields is therefore needed.

The successful synthesis of [^14^C]-SAs on a micro-scale requires stable solvents, suitable reaction conditions, and simple purification methods [23]. The conventional method of synthesizing unlabeled SAs consists of four steps: acetylation of aniline using acetic anhydride, chlorosulfonation of *N*-acetylaniline with ClSO_3_H, condensation of sulfonyl chloride with nucleophiles such as amines, and alkaline hydrolysis of the acetyl-protecting group [24–27]. However, the synthetical conditions are suitable for obtaining SAs in gram amounts and cannot be down-scaled to synthesize SAs in milligram quantities, due to the difficulty of mixing under solvent-free conditions and the crystallization of the products. In a previous study, [^14^C]-SDZ labeled on the heterocyclic ring was prepared via the reaction of *N*-acetylsulfanilyl chloride with [^14^C]-2-aminopyrimidine [28]. By contrast, [^14^C]-labeling of the phenyl ring of SDZ and other common SAs (such as SMM and SMX), required to trace the transformation of phenyl ring of SAs, has yet to be accomplished.

In this study, we report methods for preparation of typical SAs with [^14^C]- or [^13^C]-labeling of the phenyl ring with good yields, especially the synthesis of [^14^C]-labeled SAs on a micro-scale (milligram-level). These methods can be employed to prepare a variety of [^14^C]- or [^13^C]-labeled SAs.

## Materials and methods

### Chemicals

**Fig. 1:**
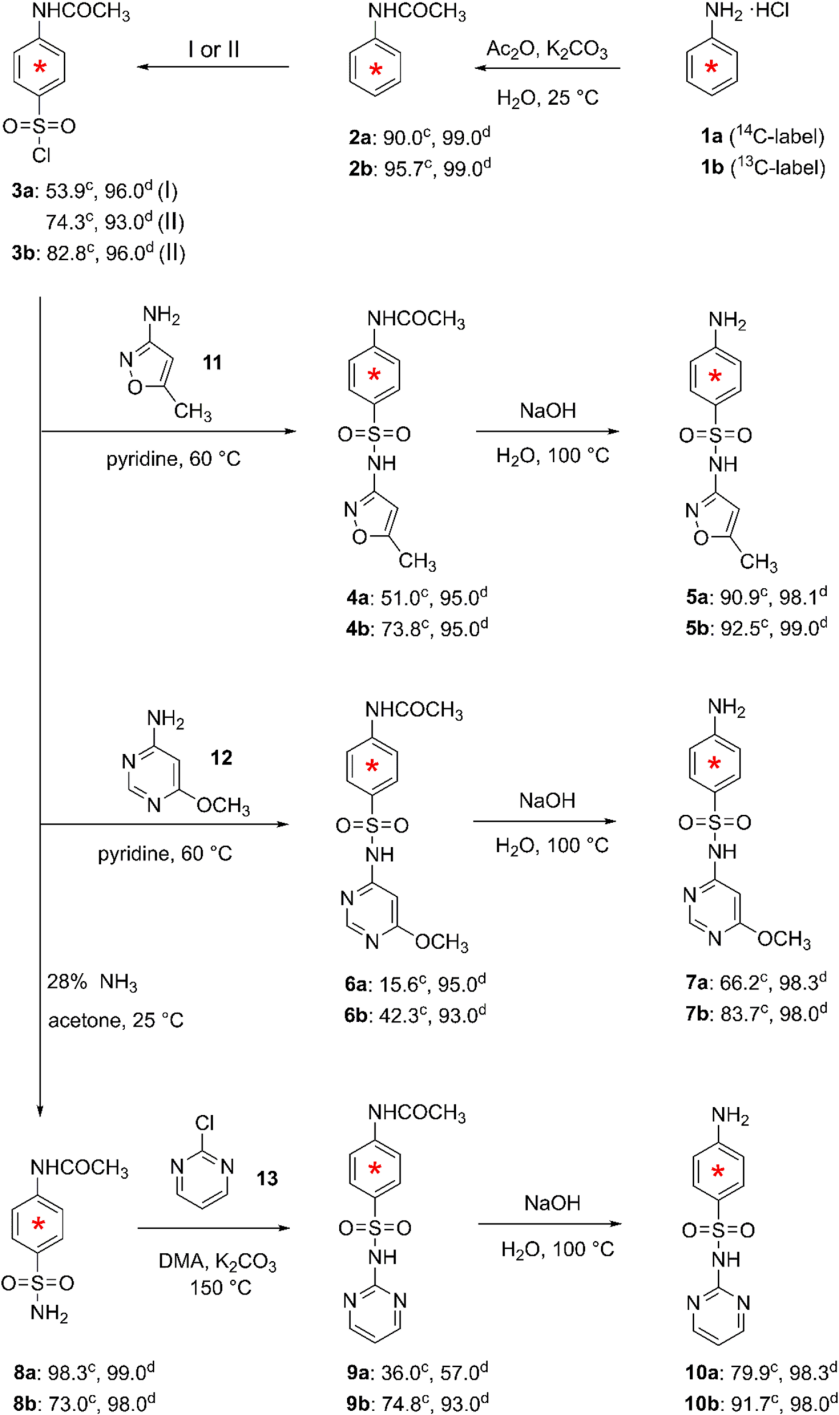
Synthetic pathways of [^14^C]- and [^13^C]-labeled SMX (**5a**, **5b**), [^14^C]- and [^13^C]-labeled SMM (**7a**, **7b**), and [^14^C]- and [^13^C]-labeled SDZ (**10a**, **10b**). **I** Method: ClSO_3_H + NaCl in CCl_4_, 58 °C; purification by flash column chromatography. **II** Method: ClSO_3_H + SOCl_2_ in CCl_4_, 58 °C; without purification. Yields% (^c^) and purities% (^d^) of the synthesized [^14^C]- or [^13^C]-labeled compounds are provided. The radiochemical purity of the [^14^C]-labeled intermediates and [^14^C]-labeled SAs was determined by TLC coupled to autoradiography and by HPLC coupled to LSC, respectively. The chemical purity of the [^13^C]-labeled intermediates and SAs was determined by HPLC

Uniformly [phenyl-ring-^14^C]-labeled aniline hydrochloride (**1a**, Fig. 1, 2.96 × 10^9^ Bq/mmol, 99% radiochemical purity) and uniformly [phenyl-ring-U-^13^C]-labeled aniline hydrochloride (**1b**, Fig. 1, 99% of ^13^C atom, 98% chemical purity) were purchased from Moravek Inc. (California, USA) and Alsachim (Illkirch Graffenstaden, France), respectively. Unlabeled SDZ, SMM, SMX, *N*-acetylaniline, and *N*-acetylsulfanilyl chloride (purity ≥ 99%) were purchased from J&K Co. (Shanghai, China). All other reagents were obtained from Nanjing Chemical Reagent Co., Ltd. (Nanjing, China) and were of analytical purity grade. Pyridine was dried over a 4 Å molecular sieves for at least 48 h prior to its use.

### Analyses

The reaction products were purified by flash column chromatography (CHEETAH TMMP100; Agela, Tianjin, China) or preparative thin-layer chromatography (TLC) on preparative silica gel plates (GF254, 1 mm, 20 × 20 cm; Huanghai, Shandong, China). The purity of the products was analyzed on analytical silica gel plates (GF254, 0.25 mm, 3 cm × 10 cm, Huanghai, Shandong, China) by analytical TLC coupled to an imaging scanner (Typhoon Trio^+^; GE Healthcare, U.S.), or by high-performance liquid chromatography (HPLC, 1100 system; Agilent Technology, USA). The synthesized products were identified on an HPLC system (1260; Agilent Technology, USA) coupled to a Q-TOF tandem mass spectrometer (HPLC-Q-TOF–MS/MS, triple TOF 5600 system; AB SCIEX, USA) and by NMR spectroscopy (AVANCE III HD-500; Bruker, Germany). Radioactivity was determined by liquid scintillation counting (LSC, LS6500; Beckman Counter, USA). The specific activities of **5a**, **7a**, and **10a** were calculated from the chemical masses determined by HPLC and the amounts of radioactivity determined by LSC. Details of the instruments used in the purifications and analyses are provided in the Additional file 1.

### Syntheses

#### Synthesis of [^14^C]-SMX (5a) and [^14^C]-SMM (7a)

##### Uniformly [phenyl-ring-^14^C]-labeled *N*-acetylaniline (2a)

K_2_CO_3_ powder (128 mg, 0.93 mmol) and acetic anhydride (66 μL, 0.70 mmol) were sequentially added to [^14^C]-labeled aniline hydrochloride (**1a**, 3.70 × 10^8^ Bq, 2.96 × 10^9^ Bq/mmol, 99.0% purity, 0.125 mmol) in deionized water (10 mL) at 25 °C (Fig. 1). The mixture was further stirred at 25 °C for 1 h and then extracted five times with ethyl acetate (10 mL each). The extract was dried with anhydrous Na_2_SO_4_ and evaporated under vacuum to ~ 1 mL. The product in the extract was purified by flash chromatography (for details, see Additional file 1: SI.1) with an elution gradient (Additional file 1: Table S1), resulting in **2a** (3.33 × 10^8^ Bq, 2.96 × 10^9^ Bq/mmol) at 90.0% yield. TLC, performed using petroleum ether:ethyl acetate (1:4, v:v) containing 0.2% CH_3_COOH as the eluent (*R*_f_ = 0.45), coupled to autoradiography (Additional file 1: SI.2) showed a radiochemical purity of 99.0%.

##### Uniformly [phenyl-ring-^14^C]-labeled *N*-acetylsulfanilyl chloride (3a)

ClSO_3_H (45 μL, 0.67 mmol) was added dropwise with stirring in an ice bath to **2a** (2.59 × 10^8^ Bq, 2.96 × 10^9^ Bq/mmol, 0.09 mmol, 99.0% purity) in CCl_4_ (1 mL). The mixture was further stirred at 58 °C for 2 h, after which NaCl (4 mg, 0.07 mmol) was added (Fig. 1, Method I). Following another 2 h of stirring, the mixture was cooled to room temperature. After hydrolysis of the residual chlorosulfonic acid with ice-cold water (10 mL), the mixture was extracted twice with ethyl acetate (35 mL each). The extracts were dried with anhydrous Na_2_SO_4_ and evaporated to ~ 1 mL. The product in the extract was purified using flash chromatography (Additional file 1: SI.1) with an elution gradient (Additional file 1: Table S1), resulting in **3a** (1.39 × 10^8^ Bq, 2.96 × 10^9^ Bq/mmol) at 53.9% yield. TLC, performed using petroleum ether:ethyl acetate (1:4, v: v) containing 0.2% CH_3_COOH as the eluent (*R*_f_ = 0.35), coupled to autoradiography (Additional file 1: SI.2) showed a radiochemical purity of 96.0%.

##### Uniformly [phenyl-ring-^14^C]-labeled *N*-acetylsulfamethoxazole (4a) and uniformly [phenyl-ring-^14^C]-labeled *N*-acetylsulfamonomethoxine (6a)

Synthesis of **4a**: **3a** (4.07 × 10^5^ Bq, 2.96 × 10^9^ Bq/mmol) in 200 uL of acetone was first diluted with unlabeled **3** (15 mg, 0.064 mmol). Next, 3-amino-5-methylisoxazole (**11**, 13 mg, 0.130 mmol), anhydrous pyridine (11 μL, 0.130 mmol), and five pieces of molecular sieve (4 Å, diameter: 1 mm) were sequentially added with stirring in an ice bath. After another 5 h of stirring at 60 °C, the mixture was diluted with methanol (200 μL) and separated using preparative TLC, with petroleum ether: ethyl acetate (1:4 / v: v) containing 0.2% CH_3_COOH as the eluent. The product band of **4a** (*R*_f_ = 0.53) was scraped from the TLC plate and extracted six times with ethyl acetate (15 mL each). Concentration of the extract by evaporation yielded **4a** (2.07 × 10^5^ Bq, 7.40 × 10^6^ Bq/mmol, 51.0% yield) with 95.0% purity as determined by TLC coupled to autoradiography (Additional file 1: SI.2).

Synthesis of **6a**: **3a** (3.7 × 10^7^ Bq, 2.96 × 10^9^ Bq/mmol) in 200 uL of acetone was first diluted with unlabeled **3** (11.7 mg, 0.05 mmol), followed by the sequential addition of 4-amino-6-methoxypyrimidine (**12**, 16.1 mg, 0.129 mmol), anhydrous pyridine (10 μL, 0.124 mmol), and five pieces of molecular sieve (4 Å, diameter: 1 mm) with stirring in an ice bath. After an additional 23 h of stirring at 60 °C, **6a** (*R*_f_ = 0.18) was purified as described for **4a**. The purity of **6a** (5.77 × 10^6^ Bq, 7.55 × 10^8^ Bq/mmol, 15.6% yield) was 95.0% as determined by TLC coupled to autoradiography (Additional file 1: SI.2).

##### Uniformly [phenyl-ring-^14^C]-labeled SMX (5a) and uniformly [phenyl-ring-^14^C]-labeled SMM (7a)

**4a** (2.04 × 10^5^ Bq, 7.40 × 10^6^ Bq/mmol) or **6a** (4.81 × 10^6^ Bq, 7.55 × 10^8^ Bq/mmol) was heated in NaOH solution (10%, 1 mL) for 3 h at 100 °C and then neutralized with 6 M HCl to pH 6. The products were extracted eight times with ethyl acetate (15 mL each). The extracts were dried with anhydrous Na_2_SO_4_, evaporated to ~ 0.5 mL, and purified by preparative TLC using petroleum ether: ethyl acetate (1:4/v:v) containing 0.4% CH_3_COOH as the eluent. The product band of **5a** or **7a** (*R*_f_ = 0.6 or 0.51, respectively) was scraped from the plates and extracted six times with ethyl acetate (15 mL each). The extracts were evaporated to ~ 0.1 mL, resulting in **5a** (1.85 × 10^5^ Bq, 7.40 × 10^6^ Bq/mmol) or **7a** (3.18 × 10^6^ Bq, 7.55 × 10^8^ Bq/mmol) at 90.9% or 66.2% yield, respectively, and purities of 98.1% or 98.3%, respectively, as determined by HPLC (Additional file 1: SI.4). The chemical structures of **5a** and **7a** were elucidated by ^1^H-NMR, ^13^C-NMR (Additional file 1: SI.5), and LC-Q-TOF–MS/MS (Additional file 1: SI.6) using the corresponding unlabeled compounds synthesized according to the same procedures.

#### Synthesis of [^14^C]-SDZ (10a)

##### Uniformly [phenyl-ring-^14^C]-labeled *N*-acetylsulfanilyl chloride (3a)

**2a** (1.11 × 10^9^ Bq/mmol, 99.0% purity) was synthesized from **1a** (3.70 × 10^8^ Bq, 2.96 × 10^9^ Bq/mmol, 99.0% purity, 0.125 mmol) diluted with unlabeled **1** (26.6 mg, 0.205 mmol) using the method described above. The yield was 90%.

Next, **2a** (2.48 × 10^8^ Bq, 0.22 mmol) in CCl_4_ (0.5 mL) was diluted with unlabeled *N*-acetylaniline (23.5 mg, 0.17 mmol). ClSO_3_H (170 μL, 2.52 mmol) was added dropwise with stirring in an ice bath. After the mixture had been stirred at 58 °C for 2 h, SOCl_2_ (25 μL, 0.34 mmol) was added. The resulting solution was heated for another 2 h at 58 °C (Fig. 1, Method II), cooled to room temperature, and extracted twice with ethyl acetate (35 mL each). The extracts were dried with anhydrous Na_2_SO_4_ and evaporated to ~ 1 mL. The purity of the product, **3a** (1.98 × 10^8^ Bq), in the mixture was 93.0%, as determined by TLC, using petroleum ether:ethyl acetate (1:4 / v: v) containing 0.2% CH_3_COOH as the eluent (*R*_*f*_ = 0.35), coupled to autoradiography. The mixture without purification was directly used for the synthesis of **8a**. The yield of **3a** according to its purity in the mixture was 74.3%.

##### Uniformly [phenyl-ring-^14^C]-labeled *N*-acetylsulfonamide (8a)

Crude **3a** (1.98 × 10^8^ Bq, 6.29 × 10^8^ Bq/mmol, 93.0% purity) was mixed with acetone (1 mL), after which ammonium hydroxide (0.5 mL, 28% NH_3_ in water) was added dropwise at 0 °C. The mixture was vigorously stirred at room temperature for 1 h and the pH was adjusted to 6 with 6 M HCl. It was then extracted eight times with ethyl acetate (15 mL each), dried with anhydrous Na_2_SO_4_, and evaporated. The product was purified by flash chromatography with an elution gradient (Additional file 1: Table S1), resulting in **8a** (1.81 × 10^8^ Bq) with a 98.3% yield and a purity of 99.0% as determined by TLC, using petroleum ether:ethyl acetate (1:4/v:v), containing 0.2% CH_3_COOH as eluent, (*R*_*f*_ = 0.26) coupled to autoradiography.

##### Uniformly [phenyl-ring-^14^C]-labeled *N*-acetylsulfadiazine (9a)

2-Chloropyrimidine (**13**, 48.7 mg, 0.42 mmol) and K_2_CO_3_ (58.6 mg, 0.42 mmol) were added sequentially to **8a** (1.74 × 10^8^ Bq, 6.29 × 10^8^ Bq/mmol, 0.28 mmol, 99.0% radiochemical purity) in *N*,*N*-dimethylacetamide (DMA, 800 μL) with stirring at room temperature. The mixture was heated at 150 °C for 4.5 h, and the solvent *N*,*N*-dimethylacetamide was then removed by evaporation. The crude product was dissolved in water, cooled in an ice bath, and the pH was adjusted to 6 with 6 M HCl. The precipitate, comprising **9a** (1.10 × 10^8^ Bq), was washed with ice-cold water. The purity of the product was 57%, as determined by TLC (Additional file 1: SI.2), using petroleum ether:ethyl acetate (1:4/v:v) containing 0.2% CH_3_COOH as the eluent, coupled to autoradiography (*R*_f_ = 0.13). The yield of **9a** according to its purity was 36.0%.

##### Uniformly [phenyl-ring-^14^C]-labeled SDZ (10a)

Crude **9a** (9.25 × 10^7^ Bq, 6.29 × 10^8^ Bq/mmol, 57.0% radiochemical purity) was reacted with NaOH solution (10%, 5 mL) for 3 h at 100 °C and neutralized with 6 M HCl to pH 6. The product was extracted eight times with ethyl acetate (15 mL each). The extract was dried with anhydrous Na_2_SO_4_, evaporated to ~ 0.5 mL, and the crude product then recrystallized from boiling methanol. The precipitate was centrifuged and washed three times with methanol, resulting in **10a** (3.11 × 10^7^ Bq, 6.29 × 10^8^ Bq/mmol). The purity was 98.3% as determined by HPLC (*t*_R_ = 5.73 min. For details, see Additional file 1). The supernatant was further extracted five times with ethyl acetate (15 mL each). The extract was dried with anhydrous Na_2_SO_4_ and evaporated to dryness, resulting in solids containing **10a**. These were mixed with unlabeled SDZ (54 mg) and then recrystallized from boiling methanol. The precipitate was washed three times with methanol, resulting in another portion of **10a** with a low specific activity (1.10 × 10^7^ Bq, 7.40 × 10^7^ Bq/mmol) and a radiochemical purity of 98.3%. The total amount of **10a** was 4.21 × 10^7^ Bq, with a total yield of 79.9%. The chemical structure of **10a** was elucidated by ^1^H-NMR, ^13^C-NMR (Additional file 1: SI.5), and LC-Q-TOF–MS/MS (Additional file 1: SI.6) using the corresponding unlabeled compounds synthesized according to the same procedures.

#### Synthesis of [^13^C]-SMX (5b), [^13^C]-SMM (7b), and [^13^C]-SDZ (10b)

##### Uniformly [phenyl-ring-^13^C]-labeled *N*-acetylaniline (2b)

K_2_CO_3_ (0.32 g/mL, 30 mL, 69 mmol) and acetic anhydride (4.70 g, 126 mmol) were added sequentially to [^13^C]-labeled aniline hydrochloride (**1b**, 3.00 g, 23 mmol, 99% of ^13^C atom) in a 200-mL flask with stirring at 25 °C. The mixture was further stirred at 25 °C for 1 h and then extracted five times with ethyl acetate (15 mL each). The extract was washed with 20 mL of H_2_O and then evaporated, resulting in **2b** (3.01 g, 99% of ^13^C atom, 99.0% purity) with a yield of 95.7% (for details, see Additional file 1: SI.4).

##### Uniformly [phenyl-ring-^13^C]-labeled *N*-acetylsulfanilyl chloride (3b)

ClSO_3_H (19.8 g, 170 mmol) was added dropwise to **2b** (3.00 g, 22 mmol, 99% of ^13^C atom) in CCl_4_ (5 mL) with stirring in an ice bath. After further stirring at 58 °C for 2 h, SOCl_2_ (2.67 g, 22 mmol) was added (Fig. 1, Method II). The mixture was heated at 58 °C for another 2 h and cooled to room temperature. The dropwise addition of ice-cold water (10 mL) to the mixture induced the formation of white crystals, which were washed twice with ice-cold water (each 10 mL) by filtration. The yield of the resulting product, **3b** (4.29 g, 99% of ^13^C atom, 96.0% purity) (Additional file 1: SI.4), was 82.8%.

##### Uniformly [phenyl-ring-^13^C]-labeled *N*-acetylsulfamethoxazole (4b), *N*-acetylsulfamonomethoxine (6b), and *N*-acetylsulfadiazine (9b)

Synthesis of **4b**: 3-amino-5-methylisoxazole (**11**, 412 mg, 4.2 mmol), anhydrous pyridine (339 μL, 4.2 mmol), and 10 pieces of molecular sieve (4 Å, diameter: 1 mm) were added sequentially to **3b** (500 mg, 2.1 mmol, 99% of ^13^C atom) in acetone (2 mL) with stirring in an ice bath. The mixture was further stirred for 7 h at 60 °C. After removal of the molecular sieves, evaporation of the acetone, and crystallization in ice-cold water, the crude product **4b** (470 mg, 99% of ^13^C atom, 95.0% purity) (Additional file 1: SI.4) was obtained at 73.8% yield.

Synthesis of **6b**: 4-amino-6-methoxypyrimidine (**12**, 526 mg, 4.2 mmol), anhydrous pyridine (339 μL, 4.2 mmol), and 10 pieces of molecular sieve (4 Å, diameter: 1 mm) were added to **3b** (500 mg, 2.1 mmol, 99% of ^13^C atom) in acetone (2 mL) with stirring in an ice bath. The reaction conditions and workup were as described for the synthesis of **4b**. The yield of the crude product **6b** (286 mg, 99% of ^13^C atom, 93.0% purity) (Additional file 1: SI.4) was 42.3%.

Synthesis of **8b**: Ammonium hydroxide (5 mL, 28% NH_3_ in water) was mixed vigorously with **3b** (1.0 g, 4.3 mmol, 99% of ^13^C atom) in acetone (10 mL) in an ice bath. After 1 h of stirring at 25 °C, the acetone was removed by evaporation, ice-cold water was added, and the pH was adjusted to  6 with 6 M HCl. Filtration and washing of the mixture with ice-cold water resulted in **8b** (672 mg, 99% of ^13^C atom, 98.0% purity) (Additional file 1: SI.4) with a yield of 73.0%.

Synthesis of **9b**: 2-chloropyrimidine (**13**, 361 mg, 3.1 mmol) and K_2_CO_3_ (439 mg, 3.2 mmol) were added with stirring at room temperature to **8b** (450 mg, 2.1 mmol, 99% of ^13^C atom) in *N*,*N*-dimethylacetamide (3.5 mL). After 5 h of stirring at 150 °C, the mixture was processed as described above for the synthesis of **9a**. The resulting product, **9b** (494 mg, 99% of ^13^C atom, 93.0% purity) (Additional file 1: SI.4), was obtained in 74.8% yield.

##### Uniformly [phenyl-ring-^13^C]-labeled SMX (5b), SMM (7b), and SDZ (10b)

**4b** (300 mg, 99% of ^13^C atom), **6b** (280 mg, 99% of ^13^C atom), and **9b** (350 mg, 99% of ^13^C atom) were individually hydrolyzed in NaOH solution (10%, 3 mL) for 3 h at 100 °C, then neutralized to pH 6 with 6 M HCl and cooled in an ice bath. The precipitates were washed with ice-cold water six times (1 mL each) and dissolved in boiling methanol (SAs:methanol = 1:1, w:v). The methanol solutions were cooled in an ice bath to recrystallize the products, which were then separated by centrifugation (10 min, 2810 g) and washed twice with ice-cold methanol, resulting in **5b** (238 mg, 99.0% purity), **7b** (204 mg, 98.0% purity), and **10b** (276 mg, 98.0% purity) in 92.5%, 83.7%, and 91.7% yield, respectively(Additional file 1: SI.4).

## Results and discussion

SMX, SMM, and SDZ with uniform ^13^C and ^14^C labeling on the phenyl ring were prepared from commercially available, labeled aniline in a four-step or five-step synthesis (Fig. 1). The yields and radiochemical or chemical purities of the products are reported in Fig. 1. Three unlabeled SAs and the respective intermediates were similarly synthesized and characterized using HPLC-Q-TOF–MS/MS, and NMR (Additional file 1: Table S2).

### Synthesis of [^14^C]- or [^13^C]-SMX, [^14^C]- or [^13^C]-SMM, and [^14^C]- or [^13^C]-SDZ

Chlorosulfonation of aniline on the *para*-position of the amino group using ClSO_3_H was the key step in the synthesis of SAs. Prior to this step, the aniline was acetylated to prevent possible oxidation of the amino group and *bis*-sulfonation on the ring during chlorosulfonation. The acetylation was performed in aqueous solution, and K_2_CO_3_ was added to improve nucleophilic activity of aniline (**1a**), resulting in acetylaniline (**2a**), with a good yield of 90.0%. Our preparation of **2a** with fewer procedures was more convenient than the previously reported method [29].

Chlorosulfonation of **2a** with ClSO_3_H generated the key intermediate **3a**, a precursor in the synthesis of a variety of [^14^C]-SAs labeled on the phenyl ring via reactions with different amino heterocycles and subsequent alkaline hydrolysis. In a previous study, 1.1 g of **2a** at a high molar ratio of ClSO_3_H to **2a** (18:1) was used to obtain **3a**, which formed a white solid after crystallization in water [29]. However, this method of using a high volume of ClSO_3_H cannot be applied to synthesize **3a** at milligram scale (12 mg of **2a**), because the hot H_2_SO_4_, derived from the hydrolysis of excess ClSO_3_H in water, causes the decomposition of **3a**, resulting in a very low yield. HPLC-Q-TOF–MS/MS also showed the conversion of a large amount of **3a** to *N*-acetylsulfanilic acid (data not shown). In addition, the solvent-free condition used in previous studies may result in an inhomogeneous mixture of the reactants at a micro-scale. Nguyen-Hoang-Nam et al. [28] also found that it was difficult to synthesize sulfonyl chloride in a small amount and thus failed to obtain micro-quantities (100 mg) of *N*,*N*-di(2-chloro-*n*-propyl)aminobenzenesulfonyl chloride labeled on the phenyl ring by chlorosulfonation with ClSO_3_H and the corresponding [^14^C]-sulfonamide derivatives. Therefore, we used a low molar ratio of 1:7.4 in solvent CCl_4_, and added NaCl to the reaction mixture to consume the by-product H_2_SO_4_. These modifications not only completely converted **2a**, they also reduced the decomposition of **3a** to *N*-acetylsulfanilic acid by hot H_2_SO_4_, such that **3a** was produced in a good yield (53.9% after purification; Fig. 1).

Water inhibits the condensation of **3a** with amino heterocyclic compounds (e.g., **11** and **12**). In our method, water interference was avoided by the inclusion of molecular sieves to adsorb the water during the condensation. With this method, **4a** and **6a** were obtained in yields of 51.0% and 15.6%, respectively (Fig. 1).

The condensation of **3** with amino heterocycles involved a nucleophilic substitution. Compound **11** had a higher nucleophilic activity than compound **12**, according to their electron cloud density, which was in agreement with the higher yield of **4a** than **6a** (51.0% vs. 15.6%) and of **4b** than **6b** (73.8% vs. 42.3%) (Fig. 1). The condensation of **3** with other heterocyclic compounds can be used to synthesize other [^14^C]- or [^13^C]-labeled sulfonamides, such as the synthesis of SDZ from 2-aminopyrimidine [28]. However, owing to the low nucleophilic activity of 2-aminopyrimidine, the yield of **10a** at micro-scale was very low (7.4%; overall yield of **10a** from **1a**: 2.4%) and the yield of **9b** (21.0%) was lower than that of either **4b** (73.8%) or **6b** (42.3%) (Fig. 1). Therefore, **10a** and **10b** were synthesized in a five-step synthetic pathway (Fig. 1), in which two steps were used to synthesize **9** instead of one step. First, **8** was synthesized by the condensation of **3** with ammonium hydroxide, which has high nucleophilic activity and is a base capable of neutralizing the by-product H_2_SO_4_. Good yields were achieved for both **8a** (98.3%) and **8b** (73.0%). The coupling of **8** to **13** produced both **9a** and **9b** in good yields of 36.0% and 74.8%, respectively. The synthesis of **9** from **3** via this two-step pathway not only completely converted **3** to **8** with a high stability, thus avoiding the decomposition of **3**, it also resulted in a much higher overall yield than the one-step reaction (35.4% vs. 7.4% for **9a**, 54.6% vs. 21.0% for **9b**).

### ^13^C-NMR of [^13^C]-SMX, [^13^C]-SMM, and [^13^C]-SDZ

**Fig. 2:**
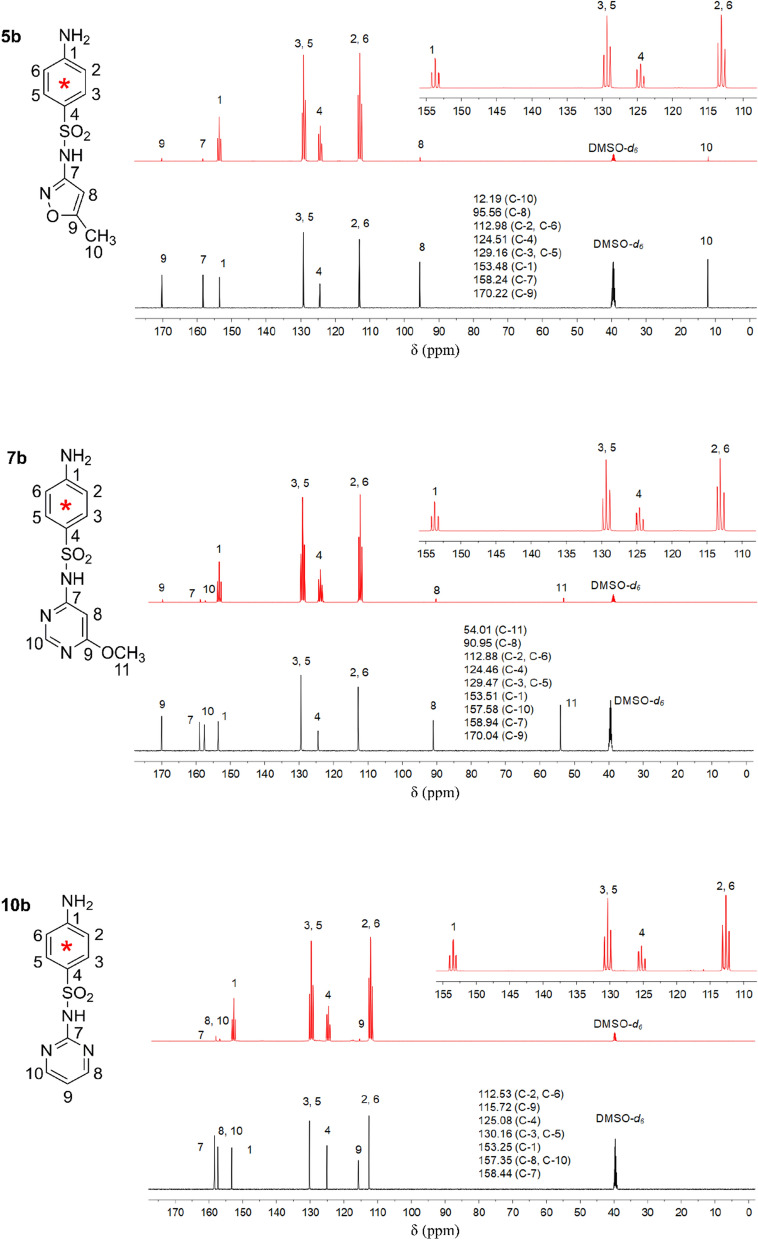
^13^C-NMR spectra of [^13^C]-SMX (**5b**), [^13^C]-SMM (**7b**), and [^13^C]-SDZ (**10b**). The positions of the numbered C-atoms are given in the corresponding structure of the [^13^C]-labeled SAs. The red and black lines represent the spectra of SAs with [^13^C]-labeling or natural ^13^C-abundance. The signal of the C-atoms at the [^13^C]-labeled benzene ring is enlarged. The chemical shifts of the numbered C-atoms with natural abundance are listed

The ^13^C-NMR spectra of three [^13^C]-SAs and their corresponding unlabeled compounds are shown in Fig. 2. The significant triplet signals allowed the assignment of the signals at 112.53–112.98 ppm, 124.46–125.08 ppm, 129.16–130.16 ppm, and 153.25–153.51 ppm to ^13^C-atoms of the benzene ring. ^13^C-tracers can provide more structural information about the fate and behaviors of labeled C-atoms in environmental matrixes than obtained with radioactive [^14^C]-tracers [30]. The peaks of the C-atoms in ^13^C-labeled compounds are split into triplets due to ^13^C–^13^C coupling and they have a much higher intensity than those in non-labeled compounds containing ^13^C-atoms in natural abundance (1.1%). Accordingly, the triplet signals can be used to identify the chemical nature of labeled carbon atoms, as demonstrated for the residues of pesticides (e.g., cyprodinil), humus monomers (e.g., catechol), and emerging pollutants (e.g., tetrabromobisphenol A) bound to soil humic substances [31–33], thus providing unambiguous information about the incorporation of pollutants (e.g., SDZ, nonylphenol, and chlorophenol) into humic substances [34–36].

### Advantages of the synthetic methods

The main advantage of our synthetic methods over those previously reported is the ability to synthesize [^14^C]-SAs at micro-scale using commercially available, relatively inexpensive [^14^C]-labeled **1** (~ 16.2 mg). Unlike the classic synthetic pathway, which proceeds via the condensation of **3** with aminoheterocycles, in our reaction the pathway that includes the condensation of **8** with chloroheterocylces has been optimized for the synthesis of [^14^C]-labeled SAs using an aminoheterocycle of low nucleophilic activity or of high steric hindrance, either of which results in [^13^C]-labeled SAs in good yields.

Purification is important for product quality. The methods for purification described herein are appropriate for [^14^C]-compounds produced in small amounts as they result in a high purity. Both crystallization in water, as a purification procedure, and the direct use of the reaction mixture without further purification are applicable to the synthesis of unlabeled SAs at a gram scale [26, 27], but not to the synthesis of [^14^C]-labeled SAs at a milligram scale, because impurities may affect the next reaction in the absence of purification, but recrystallization may result in the recovery of smaller amounts of product. In this study, we used classic chromatographic separation methods, such as flash column chromatography and preparative TLC, to purify small amounts of [^14^C]-products.

## Conclusions

This study describes optimized methods for the synthesis of SAs labeled with ^14^C or ^13^C on the phenyl ring using commercially available [^14^C]- or [^13^C]-aniline, especially the synthesis of [^14^C]-labeled SAs on a micro-scale (milligram amounts). Three typical sulfonamide antibiotics, SMX, SMM, and SDZ, with [^14^C]- or [^13^C]-labeling were prepared in good yields (5.0–22.5% for ^14^C, 28.1–54.1% for ^13^C relative to aniline). Both four-step (via the condensation of **3** and aminoheterocycles) and five-step (via the condensation of **8** and chloroheterocycles) reactions were examined. The four-step pathway is suitable for the synthesis of large amount of SAs (e.g., grams) or SAs containing aminoheterocyles of high nucleophilic activity, and the five-step pathway for the synthesis of SAs (e.g*.*, SDZ) in milligram amounts and containing an aminoheterocycle of low nucleophilic activity. Both can be employed to prepare commercially unavailable labeled SAs for use in studies on the fate and behavior of these drugs in natural and engineered environments and biological systems.

## Supplementary Information

**Additional file 1.** The details of the instruments and analytical methods are provided. **Table S1.** Purification of [^14^C]-labeled intermediates and SAs.** Table S2. **^1^H-NMR, ^13^C-NMR, and HPLC−MS/MS analyses of synthesized unlabeled intermediates and SAs.

## Data Availability

The complete dataset of this study is included within the article and the Additional file.

## Abbreviations

SAs: Sulfonamide antibiotics
SMX: Sulfamethoxazole
SMM: Sulfamonomethoxine
SDZ: Sulfadiazine
NERs: Non-extractable residues
NMR: Nuclear magnetic resonance
TLC: Thin-layer chromatography
HPLC: High-performance liquid chromatography
LSC: Liquid scintillation counting
MS: Mass spectrometer
*R*_*f*_: Retardation factor
*t*_*R*_: Retention time
DMA: *N*,*N*-Dimethylacetamide
