# Supplementary material for: Synthesis of typical sulfonamide antibiotics with [14C]- and [13C]-labeling on the phenyl ring for use in environmental studies
Source: Environ Sci Eur. 2022 Mar 8;34(1):23. doi: 10.1186/s12302-022-00598-z (PMC8904343; doi:10.1186/s12302-022-00598-z)
Supplement: Supplementary file 1 — Additional file 1. The details of the instruments and analytical methods are provided. Table S1. Purification of [14C]-labeled intermediates and SAs. Table S2. 1H-NMR, 13C-NMR, and HPLC−MS/MS analyses of synthesized unlabeled intermediates and SAs. [file 12302_2022_598_MOESM1_ESM.docx]

**Supporting Information**

**Synthesis of typical sulfonamide antibiotics with [^14^C]- and [^13^C]-labeling on the phenyl ring for use in environmental studies**

Xuan Wu^1,2^, Yao Yao^1^, Lianhong Wang^1^, Dashun Zhou^2^, Feifei Sun^1,*^, Jianqiu Chen^2^, Philippe Francois-Xavier Corvini^1,3^, Rong Ji^1,*^

1. State Key Laboratory of Pollution Control and Resource Reuse, School of the Environment, Nanjing University, Nanjing 210023, China.

2. School of Engineering, China Pharmaceutical University, Nanjing 211198, China.

3. Institute for Ecopreneurship, School of Life Sciences, University of Applied Sciences and Arts Northwestern Switzerland, Gründenstrasse 40, Muttenz CH-4132, Switzerland

Correspondence authors:

***Feifei Sun**: Tel.: +86 25 89680589, Fax: +86 25 89680589. E-mail: sff@nju.edu.cn;

***Rong Ji**: Tel.: +86 25 89680581, Fax: +86 25 89680581. E-mail: ji@nju.edu.cn;

**SI.1. Flash column chromatography**

**2a**, **3a**, and **8a** (for numbers of compounds, see **Fig.1** in the main text) were purified by flash column chromatography using a medium-pressure preparative chromatograph system (CHEETAH TMMP100; Agela, Tianjin, China) that included a prepacked silica gel column (12 g, 40–60 μm; Agela, Tianjin, China). The system was equipped with a UV detector and a fraction collector. The flow rate was 8 mL/min; UV detection was set at 230 nm and 254 nm; 30-mL fractions were collected.

**SI.2. Thin-layer chromatography (TLC)**

TLC was conducted on silica gel (60 F254) plates. Analytical TLC plates (0.25 mm, 3 cm × 10 cm, Huanghai, Shandong, China) were used for purity analysis, with petroleum ether:ethyl acetate (1:4, v:v) containing 0.2% CH_3_COOH as the eluent. The [^14^C]-labeled compounds **4a**, **5a**, **6a**, and **7a** were purified on preparative TLC plates (1 mm, 20 × 20 cm; Huanghai, Shandong, China), which were then subjected to autoradiography on an imaging scanner (Typhoon Trio^+^; GE Healthcare, USA).

**SI.3. Liquid scintillation counting (LSC)**

LSC was performed using a Beckman LS6500 (LS6500; Beckman Counter, USA) scintillation counter and Gold Star scintillation cocktail (Meridian Biotechnologies Ltd, UK). The counts were corrected for background activity by using blank controls. Counting efficiency and color quenching were corrected using external standards.

**SI.4. High-performance liquid chromatography (HPLC)**

The purity of the products and their identification were determined using an Agilent HPLC 1100 system (Agilent Technology, USA). Chromatographic separation was achieved by injecting the sample (10 μL) into an Eclipse XDB-C18 column (250 mm × 4.6 mm, 5 μm; Agilent) at 30°C with a guard column (4.6 mm × 12.5 mm, 5 μm; Agilent). The flow-rate was set at 1 mL/min. The mobile phase for the analyses of **5a** and **10a** consisted of H_2_O (A) (containing 0.3% (v/v) CH_3_COOH) and CH_3_OH (B). The elution gradient for the analysis of **5a** started with 35% B for 15 min, linearly increased to 100% B within 5 min, maintained at 100% B for 5 min, then linearly decreased to 35% B within 5 min, with a final setting of 35% B for 5 min. The elution gradient for the analysis of **10a** started with 20% B for 15 min, linearly increased to 100% B within 5 min, maintained at 100% B for 5 min, then linearly decreased to 20% B within 5 min, with a final setting of 20% B for 5 min. The mobile phase for the analysis of **7a** consisted of H_2_O (A) (containing 0.3% (v/v) CH_3_COOH) and CH_3_CN (C), run with a gradient starting at 26% C for 10 min, followed by a linear increase to 100% C within 10 min, 100% C for 5 min, a linear decrease to 26% C within 5 min, and a final setting of 26% C for 5 min. The detection wavelength was 270 nm. The eluent fractions were collected every minute and their radioactivity determined by LSC. The mobile phase used to determine the purity of the [^13^C]-intermediates and of [^13^C]-SAs consisted of H_2_O (60%) and CH_3_CN (40%) running isocratically. Purity was calculated according to the area normalization method at 270 nm.

**SI.5. Nuclear magnetic resonance (NMR) spectroscopy**

^1^H- and ^13^C-NMR spectra were recorded on an AVANCE III HD-500 spectrometer (Bruker, Germany). Samples were dissolved in CDCl_3_ or DMSO-*d*_6_ containing 1% tetramethylsilane as the internal standard.

**SI.6. High-performance liquid chromatography-mass spectrometry (HPLC-MS)**

HPLC-MS analysis was performed using an Agilent 1260 series HPLC system (Agilent Technology, USA) coupled to a Q-TOF-MS (triple TOF 5600 system; AB SCIEX, USA). An XBridge C18 column (100 mm × 2.1 mm, 3.5 μm; Waters) was thermostatically controlled at 30°C. The mobile phase used to identify synthesized unlabeled **5**, **7**, and **10** was a mixture of Milli-Q water (50%) (containing 0.1% (v/v) HCOOH) and methanol (50%) running isocratically at 0.2 mL/min. The full scan was operated in positive ESI ionization mode, with a declustering potential of 80 V and a collision energy of 10 V. The collision energy of the product scan was 25 V, and the collision energy spread 15 V. The full scan and product ion scan were operated with a mass range of *m/z* 80–800. The mobile phase for the identification of synthesized unlabeled **3** was acetonitrile (100%), and that for the other intermediates was methanol (100%) running isocratically at 0.2 mL/min. The full scan was operated in negative ESI ionization mode, with a declustering potential of −80 V and a collision energy of −10 V. The collision energy of the product scan was −35 V and the collision energy spread 15 V. The full scan and product ion scan were operated with a mass range of *m/z* 80–600. The optimal parameters for Q-TOF were as follows: nebulizer gas (N_2_) of 55 psi, heater gas (N_2_) of 55 psi, curtain gas of 35 psi, turbo spray temperature of 550°C, ion-spray voltage in ESI^+^ and ESI^−^ of 5.5 kV and −4.5 kV, respectively. The software PeakView (AB SCIEX, Foster City, CA, USA) was used in the mass spectrum analysis.

**Table S1** Purification of [^14^C]-labeled intermediates and SAs

| Compound | Purification conditions |
| --- | --- |
| **2a** | Flash column chromatography  Eluent: petroleum ether / ethyl acetate  Gradient elution program: volume fraction of ethyl acetate  0%, 5 min; 20%, 5 min; 20% to 30%, 15 min; 30%,  5 min; 50%, 5 min; 100%, 20 min  Fraction collection: 23~32 min |
| **3a** | Flash column chromatography  Eluent: *n*-hexane / ethyl acetate  Gradient elution program: volume fraction of ethyl acetate  0%, 5 min; 20%, 5 min; 20% to 30%, 10 min; 30%,  10 min; 40%, 10 min; 100%, 30 min  Fraction collection: 26~40 min |
| **4a**, **6a** | Preparative TLC  Eluent: petroleum ether / ethyl acetate (1:4 / v: v) containing 0.2% CH_3_COOH |
| **9a** | Recrystallization in water |
| **5a**, **7a** | Preparative TLC  Eluent: petroleum ether / ethyl acetate (1:4 / v: v) containing 0.4% CH_3_COOH |
| **10a** | Recrystallization in methanol |
| **8a** | Flash column chromatography  Eluent: petroleum ether / ethyl acetate  Gradient elution program: volume fraction of ethyl acetate  0%, 10 min; 0~50%, 5 min; 50%, 10 min; 50% to 70%,  10 min; 70%, 10 min; 80%, 30 min; 80% to100%,  10 min; 100%, 20 min  Fraction collection: 28~55 min |

**Table S2** ^1^H-NMR, ^13^C-NMR, and HPLC−MS/MS analyses of synthesized unlabeled intermediates and SAs

| Compound | MS fragments (m/z) | Chemical shifts (ppm)*^a^* | |
| --- | --- | --- | --- |
|  |  | ^1^H-NMR | ^13^C-NMR |
|   Unlabeled **2** | [C_8_H_8_NO]^−^, [M−H]^−^  calculated: 134.0611  detected: 134.0617  MS/MS:  92.0516 [M−C_2_H_3_O]^−^ | 7.68 (1H, s, NHCO),  7.50 (2H, d, H-2, H-6), 7.30 (2H, t, H-3, H-5),  7.10 (1H, t, H-3),  2.16 (3H, s, H-8) | /^b^ |
|   Unlabeled **3** | [C_8_H_7_ClNO_3_S]^−^, [M−H]^−^  calculated: 231.9841  detected: 231.9825  MS/MS:  168.0194 [M−SO_2_H]^−^,  126.0107 [M−C_2_H_3_O_3_S]^−^ | 10.09 (1H, s, NHCO),  8.97 (4H, s, H-2, H-3, H-5, H-6), 2.03 (3H, s, H-8). | / |
|   Unlabeled **4** | [C_12_H_12_N_3_O_4_S]^−^, [M−H]^−^  calculated: 294.0554  detected: 294.0580  MS/MS:  198.0245 [M−C_4_H_5_N_2_O]^−^, 134.0618 [M−C_4_H_5_N_2_O_3_S]^−^, 92.0514 [M−C_6_H_7_N_2_O_4_S]^−^ | 11.32 (s, 1H, SO_2_NH), 10.36 (1H, s, NHCO),  7.76 (4H, q, H-2, H-3, H-5, H-6), 6.12 (1H, s, H-8), 2.29 (3H, s, H-10),  2.07 (3H, s, H-12) | / |
| Unlabeled **5** | [C_10_H_12_N_3_O_3_S]^+^, [M+H]^+^  calculated: 254.0594  detected: 254.0595  MS/MS:  156.0108 [M−C_4_H_5_N_2_O]^+^, 108.0448 [M−C_4_H_5_N_2_O_2_S]^+^,  92.0538 [M−C_4_H_5_N_2_O_3_S]^+^ | 10.92 (1H, s, NH),  7.46 (2H, d, H-3, H-5),  6.57 (2H, d, H-2, H-6), 6.08 (3H, d, H-8, NH_2_),  2.28 (3H, s, H-10) | 170.03 (C-9), 158.09 (C-7), 153.39 (C-1), 128.97 (C-3, C-5), 124.27 (C-4), 112.74 (C-2, C-6), 95.41 (C-8),  12.12 (C-10) |
|   Unlabeled **6** | [C_13_H_14_N_4_O_4_S]^−^, [M−H]^−^  calculated: 321.0663  detected: 321.0696,  MS/MS:  134.0618 [M−C_5_H_6_N_3_O_3_S]^−^, 122.0366 [M−C_8_H_10_NO_3_S]^−^, 92.0507 [M−C_7_H_8_N_3_O_4_S]^−^ | 11.97 (1H, s, SO_2_NH), 10.34 (1H, s, NHCO),  8.40 (1H, d, H-10),  7.85 (2H, d, H-3, H-5), 7.74 (2H, d, H-2, H-6), 6.32 (1H, d, H-8),  3.83 (3H, s, H-11),  2.06 (3H, d, H-13) | / |
| Unlabeled **7** | [C_11_H_13_N_4_O_3_S]^+^, [M+H]^+^,  calculated: 281.0703,  detected: 281.0703,  MS/MS:  215.0920 [M−HSO_2_]^+^, 156.0102 [M−C_5_H_6_N_3_O]^+^, 126.0662 [M−C_6_H_4_NO_2_S]^+^, 108.0442 [M−C_5_H_6_N_3_O_2_S]^+^ | 11.36 (1H, s, NH),  8.39 (1H, d, H-10),  7.54 (2H, d, H-3, H-5), 6.58 (2H, d, H-2, H-6), 6.30 (1H, d, H-8),  6.10 (2H, s, NH_2_),  3.82 (3H, s, H-11) | 169.85 (C-9), 158.83 (C-7), 157.52 (C-10), 153.41 (C-1), 129.29 (C-3, C-5), 124.16 (C-4), 112.62 (C-2, C-6), 90.69 (C-8),  53.94 (C-11) |
|   Unlabeled **8** | [C_8_H_10_N_2_O_3_S]^−^, [M−H]^−^,  calculated: 213.0339,  detected: 231.0345,  MS/MS:  171.0236 [M−C_2_H_3_O]^−^, 134.0611 [M−H_2_NO_2_S]^−^, 92.0515 [M−C_2_H_4_NO_3_S]^−^ | 10.27 (1H, s, NH),  7.81–7.64 (4H, m, H-2, H-3, H-5, H-6),  7.24 (2H, s, NH_2_),  2.08 (3H, d, H-8). | / |
|   Unlabeled **9** | [C_12_H_12_N_4_O_3_S]^−^, [M−H]^−^  calculated: 291.0557  detected: 291.0593,  MS/MS:  227.0960 [M−C_3_HN_2_]^−^, 184.0765 [M−C_5_H_4_N_2_O]^−^, 134.0620 [M−C_4_H_4_N_3_O_2_S]^−^, 92.0262 [M−C_6_H_6_N_3_O_3_S]^−^ | 11.68 (1H, s, SO_2_NH), 10.32 (1H, s, NHCO),  8.49 (2H, d, H-8, H-10), 7.91 (2H, d, H-3, H-5), 7.73 (2H, d, H-2, H-6), 7.03 (1H, t, H-9),  2.06 (3H, d, H-13) | / |
| Unlabeled **10** | [C_10_H_11_N_4_O_2_S]^+^, [M+H]^+^,  calculated: 251.0597,  detected: 251.0584,  MS/MS:  156.0112 [M−C_4_H_4_N_3_]^+^, 108.0442 [M−C_4_H_4_N_3_OS]^+^, 92.0502 [M−C_4_H_4_N_3_O_2_S]^+^ | 11.27 (1H, s, NH),  8.48 (2H, d, H-8, H-10), 7.69–7.55 (2H, H-3, H-5), 7.00 (1H, t, H-9), 6.63-6.47 (2H, m, H-2, H-6), 6.01 (2H, s, NH_2_). | 158.37 (C-7), 157.31 (C-8, C-10), 153.17 (C-1), 130.02 (C-3, C-5), 124.97 (C-4), 115.64 (C-9), 112.35 (C-2, C-6). |

^a^ The positions of the numbered C-atoms are given in the corresponding structures.

^b^ “/”: ^13^C-NMR analysis not performed.
